# From Cancer Therapy to Winemaking: The Molecular Structure and Applications of β-Glucans and β-1, 3-Glucanases

**DOI:** 10.3390/ijms23063156

**Published:** 2022-03-15

**Authors:** Catarina Caseiro, Joana Nunes Ribeiro Dias, Carlos Mendes Godinho de Andrade Fontes, Pedro Bule

**Affiliations:** 1 CIISA—Centre for Interdisciplinary Research in Animal Health, Faculty of Veterinary Medicine, University of Lisbon, 1300-477 Lisbon, Portugal; ccaseiro@fmv.ulisboa.pt (C.C.); joananrdias@fmv.ulisboa.pt (J.N.R.D.); 2 Associate Laboratory for Animal and Veterinary Sciences (AL4AnimalS), 1300-477 Lisbon, Portugal; 3 NZYTech Genes & Enzymes, 1649-038 Lisbon, Portugal; carlos.fontes@nzytech.com

**Keywords:** β-glucans, β-glucanases, molecular structure, cancer, Dectin-1, CR3, vaccines

## Abstract

β-glucans are a diverse group of polysaccharides composed of β-1,3 or β-(1,3-1,4) linked glucose monomers. They are mainly synthesized by fungi, plants, seaweed and bacteria, where they carry out structural, protective and energy storage roles. Because of their unique physicochemical properties, they have important applications in several industrial, biomedical and biotechnological processes. β-glucans are also major bioactive molecules with marked immunomodulatory and metabolic properties. As such, they have been the focus of many studies attesting to their ability to, among other roles, fight cancer, reduce the risk of cardiovascular diseases and control diabetes. The physicochemical and functional profiles of β-glucans are deeply influenced by their molecular structure. This structure governs β-glucan interaction with multiple β-glucan binding proteins, triggering myriad biological responses. It is then imperative to understand the structural properties of β-glucans to fully reveal their biological roles and potential applications. The deconstruction of β-glucans is a result of β-glucanase activity. In addition to being invaluable tools for the study of β-glucans, these enzymes have applications in numerous biotechnological and industrial processes, both alone and in conjunction with their natural substrates. Here, we review potential applications for β-glucans and β-glucanases, and explore how their functionalities are dictated by their structure.

## 1. Introduction

β-glucans are a large class of natural polysaccharides composed of D-glucose monomers linked through β-glycosidic bonds. Although this definition technically includes β-1,4-glucans (cellulose), the term is mostly reserved for β-1,3 and mixed-linkage β-(1,3–1,4) glucans (MLGs). They are widely distributed in nature and can be found in yeast, fungi, bacteria, seaweeds and cereals, where they fulfil storage, structural and protective roles [1,2,3,4]. The molecular structure of β-glucans can vary depending on their source but, at their core, MLGs are unbranched polysaccharides of both β-1,3 and 1,4 linked glucose units, in varying proportions, whereas β-1,3 glucans consist of linear backbones of β-1,3 linked glucose units, often decorated with β-1,6 linked branches. A large amount of attention has been devoted to the molecular and structural features of β-glucans since they determine the physical properties of these polysaccharides, such as water solubility and rheological behavior, in addition to their affinity towards a variety of β-glucan interacting proteins. As such, most functional aspects of β-glucans are a consequence of backbone length (degree of polymerization, DP), the ratio of 1,3:1,4 bonds or 1,3:1,6 bonds (branching degree), interval of branching and sidechain size [5,6].

**Figure 1:**
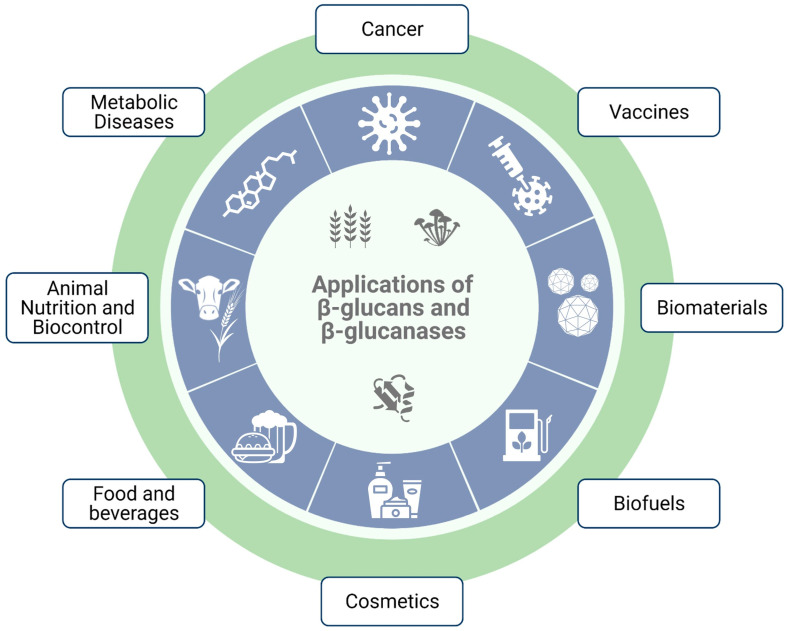
The applications of β-glucans and β-1,3-glucanases. Due to their unique physicochemical and bioactive properties, β-glucans and their processing enzymes are attractive molecules with multiple applications, ranging from cancer treatment to brewing.

In recent decades, the popularity of β-glucans as functional, bioactive and nutraceutical ingredients has steadily increased (Figure 1). Because they can interact with specific receptors present in several micro- and macro-organisms, including humans, they are able to trigger various biological responses, which can be harnessed for therapeutic purposes. The therapeutic effects of these polysaccharides can be either immunomodulatory or metabolic [5,7,8,9]. Metabolic effects, usually observed with MLGs, include modulation of the gut microbiome, improved lipidic and glycemic metabolism, and reduced cholesterol levels. Immunomodulatory effects, which are often associated with β-1,3-glucans, include anti-tumor effects, improved wound healing and alleviation of immune-related conditions. β-glucans have also been shown to be important prebiotics, improving gastrointestinal function by regulating intestinal microbiota [10,11,12,13,14,15].

Given that β-glucan receptors are present in most species, their nutraceutical applications are not restricted to humans. For instance, in animal husbandry they can be administered as a feed additive to boost immunity, reducing the need for antibiotics and improving production performances [16,17,18]. Due to their physicochemical properties, β-glucans also have a wide range of potential applications in other industries (Figure 1). The most popular uses are as a thickening and gelification agent in the food industry, and as ingredients in cosmetic and personal care products due to their soothing, moisturizing and anti-irritant properties [19,20].

β-glucan deconstruction typically results from the action of β-1,3-glucanases, including exo-1,3-β-glucosidases (EC 3.2.1.58), endo-β-1,3-glucanases (EC 3.2.1.39) and endo-1,3(4)-β-glucanases (EC 3.2.1.6). Although the first two are mostly β-1,3-glucan specific, having limited action on MLGs, endo-1,3(4)-β-glucosidases are active on both MLGs and β-1,3-glucans. This results from them being able to hydrolyze both β-1,3 and β-1,4 bonds, as long as the glucose residue whose reducing group is involved in the linkage to be hydrolyzed is itself substituted at C-3 [21,22]. They are different from lichenases or MLGases (EC 3.2.1.73), which are endo-(1,3:1,4)-β-glucosidases that can only hydrolyze β-1,4 bonds that are adjacent to a β-1,3 bond, making them MLG specific [23].

β-1,3-glucanases are widely distributed in nature and are mostly expressed by bacteria, fungi, plants and some invertebrates, where they are involved in various physiological processes [24,25]. Those include energy production, cellular remodeling and growth, defense against fungal pathogens, seed germination, digestion and reproduction [26,27,28,29]. The ability of β-glucanases to hydrolyze β-glucans down to small oligosaccharides, or even glucose, has inspired an array of biotechnological applications (Figure 1). Some popular examples for their use are: in the conversion of lignocellulosic biomass into fermentable sugars for the production of bioethanol; as feed additives to reduce the antinutritional effects of cereal β-glucans; as biocontrol agents against pathogenic fungus in various crops; to control invasive *Candida albicans* infections; as clarification agents in winemaking; to speed up germination during the malting process; and to avoid the accumulation of barley β-glucans in brewing [30,31,32,33,34].

The substrate specificities of β-glucanases, much like with all hydrolases, are intrinsically related to their tridimensional structure, the shape of their substrate-binding cleft and the constellation of substrate interacting residues. These characteristics will not only dictate substrate preferences but also the final products of digestion. Whether hydrolysis by a particular β-glucanase occurs preferentially on longer or shorter, helical or linear, or highly branched or unbranched polysaccharides, ultimately comes down to a structural compatibility between substrate and catalyst [35,36].

The purpose of this review is to provide an outline of current knowledge about β-glucans and β-glucanases, their biochemical properties, and potential biotechnological and biomedical applications. Other β-glucan interacting proteins, such as carbohydrate-binding modules (CBMs) and Dectin-1, are also briefly reviewed, as they are intrinsically related to the biological functions of β-glucans. Special focus is given to the structural features of these biomolecules and how they modulate the β-glucan:protein interaction, dictating the biological role and potential applications of β-glucans and β-1,3-glucanases.

## 2. Structure of β-Glucans

### 2.1. Variations in β-Glucan Primary Structure

Depending on the type of linkage present between their glucose monomers, β-glucans are often classified into cereal and non-cereal. Nonetheless, even within each sub-group, the primary structure of β-glucans can vary significantly depending on the source, which translates into distinct physicochemical and functional properties.

**Figure 2:**
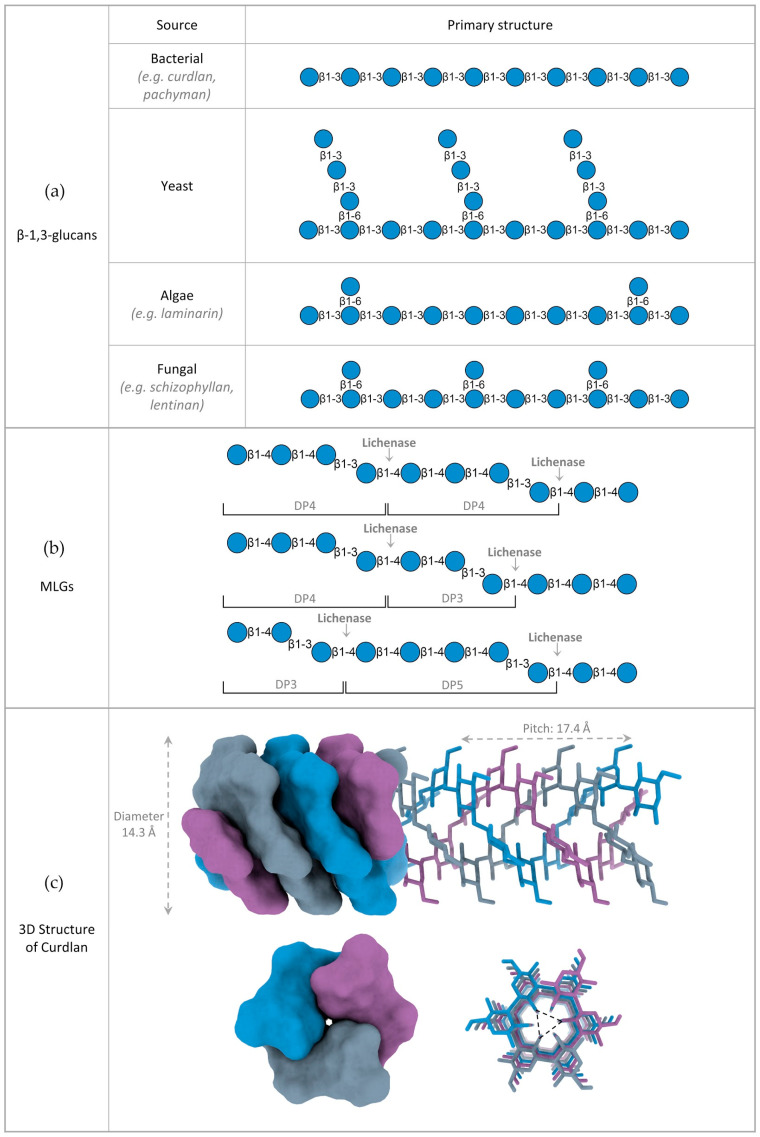
Structural features of β-glucans. (**a**) Primary structure of β-1,3-glucans from different sources. (**b**) Primary structure of mixed-linkage glucans. The arrows point towards lichenase cleavage sites. Lichenase is an endo-hydrolase that hydrolyses the MLGs into smaller oligomers. Oligosaccharide profiles in DPs vary depending on the taxonomic origin of MLGs. (**c**) Triple helical quaternary structure of curdlan. Each individual chain is represented in a different color. The pitch and diameter of the helix are represented, as well as the triangular arrangement of interstrand H-bonds.

Cereal β-glucans is a term often used for mixed-linkage glucans, which are linear and unbranched polymers composed of a mixture of β-1,3 and β-1,4 linked glucose units. The term cereal β-glucan comes from the fact that they were initially thought to be unique to the Poaceae family (grasses). However, evidence has shown that MLGs are also synthesized in other taxa, including plants from the *Equisetum* genus (horsetail), algae, lichens, fungi and even bacteria [10,37,38,39,40,41]. The ratio between β-1,4 and β-1,3 linkages varies with the species and has a significant impact on MLG’s physicochemical traits. A common tool to evaluate MLG linkage ratio, and its distribution, is the digestion with lichenases (Figure 2b). As mentioned above, lichenases are endo-(1,3:1,4)-β-glucosidases that hydrolyze β-1,4 bonds adjacent to β-1,3 linkages. Depending on the MLG’s composition, lichenase digestion will generate different amounts of end-products with different degrees of polymerization (DP), such as the trisaccharide β-glucosyl-1,4-β-glucosyl-1,3-glucose (G4G3G, DP3) or the tetrasaccharide β-glucosyl-1,4-β-glucosyl-1,4-β-glucosyl-1,3-glucose (G4G4G3G, DP4).

Generally, the most drastic differences occur between different taxonomic groups, likely suggesting different biological roles. For example, enzymatic profiling and linkage analysis of Poaceae MLG reveals a ratio of β-1,4 to β-1,3 linkages ranging from 2.2 to 2.6:1. This translates to a higher proportion of DP3 compared to DP4, with a ratio of 1.5 to 4.5:1, and the occasional occurrence of longer oligosaccharide units [42]. MLGs from horsetails, on the other hand, release mainly DP4 upon lichenase digestion. They are also abundant both on young and older regions of the stem, as opposed to Poaceae MLGs, which are largely absent in mature tissues. This suggests that *Equisetum* MLGs may have a different function in the cell wall [37,43]. No MLGs have yet been reported in green algae, although structurally similar polysaccharides that include xylose and arabinose have been isolated [44]. On the other hand, red and brown algae, which are more distantly related to plants, both have MLG in their cell walls. This suggests a convergent evolution of the (1,3;1,4)-β-glucan synthase genes. Interestingly, MLG from brown algae is composed exclusively of repeating DP3 units, which creates a highly regular structure with a tendency to self-aggregate [45]. The primary structure of lichen MLGs, often referred to as lichenan, is characterized by a higher DP3:DP4 ratio, although the ratio of β-1,4 and β-1,3 linkages varies significantly between species, ranging from 2.3:1 in *Cetraria islandica* (Iceland moss), to 0.3:1 in *Evernia prunastri* (oak moss) [46]. Besides lichens, MLGs with a high percentage of β-1,4 linked glucose have also been reported in non-lichen fungus, such as *Aspergillus fumigatus* and *Neurospora crassa* [41,47]. The occurrence of MLG has also been described in both Gram-negative and Gram-positive bacterial species, including *Sinorhizobium meliloti* and *Sarcina ventriculi* [38]. Structural profiling revealed that the MLG of *S. meliloti* consists of repeating units of the disaccharide β-glucosyl-1,3-glucose (DP2) linked by β-1,4 bonds (alternating β-1,4 and β-1,3 linkages), whereas the MLG of *S. ventriculi* is exclusively composed of repeating DP3 units, similar to that of brown algae [10,38].

The term non-cereal β-glucans is mostly used for β-1,3-glucans, which are polysaccharides with a linear backbone of β-1,3 linked glucoses, often decorated with β-1,6 linked branches. Their overall primary structure can vary significantly, depending on the length of the main chain, the interval of branching and the length of the side chains. These characteristics are mostly dependent on the source of the β-glucans, but other factors such as growth conditions, extraction and purification methods can also have a great impact on β-glucan structure [48,49]. Much like MLGs, the variations in β-1,3-glucan primary structure are more marked between different taxonomic groups. Generally, bacterial β-glucans are unbranched, yeast β-glucans have long β-1,6 linked branches, fungal β-glucans have small β-1,6 linked branches, whereas algal β-glucans are very similar to the fungal ones, but with much smaller DPs (Figure 2a). Curdlan, which was isolated from the bacteria *Alcaligenes faecalis*, is a linear and essentially unbranched β-1,3-glucan with a DP that can vary between 135 and 455 glucose units [1]. Pachyman, another example of a bacterial β-1,3-glucan, is essentially similar to curdlan, but with a smaller DP (255) and the occasional β-1,6 branch. Both of these are water insoluble. On the other hand, β-glucans extracted from several fungus, such as *Lentinus edodes* (lentinan), *Sclerotium* spp. (scleroglucan) and *Schizophyllum commune* (schizophyllan) also possess a linear β-1,3 backbone, but with β-1,6-linked monoglucose residues approximately every three or five β-1,3-linked glucose residues [50,51,52]. Unlike the bacterial β-glucans, they are mostly water soluble, suggesting that the 1,6-linked monoglucose residues have a role in defining the solubility of β-glucans. The β-glucans present in seaweeds, such as *Laminaria digitata*, *Saccharina longicruris* and *Durvillaea Antarctica*, have a β-1,3 main chain, with a small percentage of β-1,6 glycosidic bonds in the backbone and β-1,6 monoglucose side chain branching [49]. They are smaller than β-glucans from other organisms and do not have a structural function, rather serving as energy storage polysaccharides [49]. Yeast cell wall β-glucans also possess β-1,6 branching, but with a different structure. They have longer branches with additional β-1,3 linked glucoses, sometimes over 50 units long, which can form interchain linkages [49]. As such, the longer the branches, the less water soluble the β-glucans.

Besides branching, the DP of β-1,3-glucans is also a determinant factor in their water solubility, which decreases with size. As such, short branched polysaccharides such as laminarins with a DP of 20–30, are water soluble, whereas unbranched β-1,3-glucans with DPs above 36, such as curdlan, are insoluble due to the cooperative interactions between chains becoming stronger than those between the chains and water molecules [49,53]. Although longer than laminarins, many branched fungal β-1,3-glucans, such as lentinan and schizophyllan, are still water soluble. This solubility depends on the frequency of side branches. However, at high molecular weights (DP > 1000), the branching factor does not seem to compensate for the size factor; hence, large β-glucans from yeast and fungal cell walls, although branched, are water insoluble [54]. In the case of MLGs, water solubility decreases the more β-1,4 linkages are present in the molecule. The most soluble polymers comprise approximately 30% of β-1,3 linkages and 70% of β-1,4 linkages [55].

Ultimately, variations in the chemical structure of the β-glucans will dictate their tertiary structure and consequently affect their biological activities [56].

### 2.2. 3D Structure of β-Glucans

It has long been established, both by experimental studies and computer-assisted model building, that many long chain β-1,3-glucans form triple-helical structures. Oriented fiber X-ray diffraction [57], solid state ^13^C-NMR spectroscopy [58], multi-angle laser light scattering [59], fluorescence resonance energy transfer spectroscopy [60] and molecular dynamic simulations [61] suggest a triplex structure composed of parallel strands, stabilized by extensive interstrand and interhelix hydrogen bonds [56]. For example, hydrated curdlan is a triplex of right-handed, six-fold helical chains, meaning that the individual chains of curdlan are constituted by a six-glucose unit per turn. The diameter of the triplex determined from X-ray diffraction experiments was 14.4 Å and the pitch of the helix was estimated to be 17.3 Å (Figure 2c) [57]. Similar structures were also described for the fungal β-glucans schizophyllan and lentinan, albeit with small variations in pitch and diameter [62,63]. In turn, analytical ultracentrifugation and calorimetric analysis showed that short β-1,3-glucans, such as laminarin from *Laminaria digitata*, whose molecular mass is around 5000 Da, predominantly exist in a single-strand form, with approximately 5% of triple-helical structures [64].

A curdlan triple-helix model, determined by X-ray fiber diffraction, shows interchain hydrogen bonds between the 2-OH groups of each glucose arranged in a triangular pattern. Together with a contribution from hydrophobic forces, these H-bonds are the driving force behind quaternary structure formation (Figure 2c) [49,56].

Optical rotatory dispersion measurements were applied to native and partial degraded curdlan fractions to understand the required chain length for the formation of a triple helix. A DP larger than 200 (molecular mass of 32,000 Da per chain) was found to be necessary to form an ordered structure in dilute alkaline solution (0.1 M). In turn, β-glucans with DPs below 25 are soluble and take a disordered structure in both neutral and alkaline solutions [53].

It has been shown that when β-1,3-glucans are diluted in dimethyl sulfoxide (DMSO) [65], alkaline solutions (pH higher than 12) [63], or submitted to high temperatures (>135 °C) [56,66], the strength of the interchain hydrogen bonds is not sufficient to keep the triplex helix together. As such, under these conditions, β-glucan helixes tend to dissociate into random coils [56].

The capacity of the triple helices to spontaneously renature, when the thermodynamic conditions that favor helix conformation are re-established, has also been examined. For example, alkaline-treated or DMSO-denatured lentinan and schizophyllan adopt a mixture of linear, circular and branched species of triple helix when dialyzed against water [67]. Such conformational variation is not observed in native molecules, which appear as perfect linear triplexes. This may be explained by a simultaneous assembly of the self-stabilizing triple helix structure with biosynthesis, thus suppressing errors in the triplex formation [68]. β-1,3-glucan conformations are closely related with their functional properties and biological activities. Therefore, after the renaturation process, the bioactivities of β-1,3-glucans can be lost or decreased due to structural changes. This means that extraction methods can have a significant impact on β-glucans’ functional profiles [69].

## 3. β-1,3-Glucanases

β-1,3-glucanases are the main enzymes responsible for β-glucan deconstruction. They are glycoside hydrolases that catalyze the hydrolysis of β-1,3-D-glycosidic bonds and, in some cases, β-1,4-D-glycosidic bonds, in linear or partially branched glucans [70]. They can be divided into endo-glucanases and exo-glucanases, according to the location of the cleaved bonds within the polysaccharide chain. Endo-β-1,3-glucanases cleave β-1-3 glycosidic bonds randomly inside the β-glucan chain, releasing glucan oligosaccharides as main final products, whereas exo-β-1,3-glucanases sequentially release monosaccharides from the non-reducing end of the polysaccharide chain. Exo-β-1,3-glucanases normally reverse the anomeric configuration of the end product during catalysis, whereas endo-β-1,3-glucanase action typically retains the anomeric configuration (via a double-displacement mechanism). As such, some endo-β-1,3-glucanases also have the ability to catalyze the inverse reaction, or transglycosylation reaction, where the β-1,3-oligosaccharides (glycosyl donors) are transferred a glycosyl acceptor, generating new glycosidic bonds [3,71].

β-1,3-glucanases are widely distributed among higher plants, marine invertebrates, fungi, bacteria, archaea and viruses. Mainly due to the several physiological roles played by their natural substrates, the biological functions of β-1,3-glucanases are highly diverse. In bacteria, β-1,3-glucanases are mainly used to degrade polysaccharides into oligo and monosaccharides, which are then used as an energy source [25]. In higher plants, β-glucanases participate in cell differentiation, trafficking of materials through plasmodesmata, in withstanding abiotic stress, in seed development and germination, and are involved in the defense against fungal pathogens by hydrolyzing their cell walls [72]. In fungi, β-1,3-glucanases are engaged in several roles, namely, in the mobilization of β-1,3-glucans when under limited energy conditions, and in yeast cell development and differentiation, and can also act as autolytic enzymes. [70]. In animals, β-1,3-glucanases are found in marine invertebrates, specifically in the Echinodermata, Crustacea and Mollusca phyla. They are mostly present in the digestive tract of marine mollusks and crustaceans, participating in the digestion of algal biomass, and in the eggs of Echinodermata, where they are involved in embryogenesis [25,27]. In viruses, β-glucanases are involved in degrading the host cell wall during viral entry and release [73]. Although mammals do not possess the ability to express β-1,3-glucanases, they still play a determinant role in the regulation of the gastrointestinal microbiome and, consequently, on the maintenance of both gut and general health. In humans, for example, it is widely accepted that the human gut microbiota (HGM) has far-reaching effects on human health and nutrition and is even associated with some diseases, depending on its particular composition. The maintenance of a healthy HGM is dependent on the utilization of otherwise indigestible dietary fiber by individual HGM species, where β-1,3-glucans are included. Bacteroides species, in particular, are predominant autochthonous members of the healthy HGM that can utilize β-glucans as an energy source by expressing several β-1,3-glucanases, allowing them to thrive in a highly competitive ecological niche [21].

β-1,3-glucanases are proteins with a typical molecular mass of around 30 to 50 kDa, but their sizes lay in a wide range of 12 kDa (e.g., *Citrus aurantiifolia*) up to 230 kDa (e.g., *Aspergillus nidulans*), with the largest members mostly belonging to eukaryotic species [34,71,74,75,76,77]. Typical optimal pH values for β-1,3-glucanase activity are on the slightly acidic range of 5.0 to 6.5, but can range anywhere between 4.0 (e.g., fungi *Lentinula edodes* [78]) and 9.0 (e.g., armyworm *Spodoptera frugiperda* [79]). Fungal β-1,3-glucanases seem to be particularly active on lower pH values (4.0–6.0) [77,78,80]. As with most CAZymes, the optimal temperatures for β-1,3-glucanase activity fall within a wide range, with the lowest temperature values belonging to the β-1,3-glucanases of *Aspergillus fumigates* (24–40 °C) and the highest to the β-1,3-glucanases of archaea *Pyrococcus furiosus* (100–105 °C) [74,81]. Overall, the adaptation potential of archaeal enzymes to high temperatures is greater than that of bacteria, which in turn show greater tolerance for hot conditions compared to eukaryotic enzymes. On the other hand, eukaryotic enzymes are the best at adapting to cold conditions [3].

### 3.1. Classification of β-1,3-Glucanases

β-1,3-glucanases are usually classified either according to their catalytic mechanism or based on primary sequence homology and folding similarities [3]. The Nomenclature Committee of the International Union of Biochemistry and Molecular Biology (IUBMB) and the International Commission on Enzymes or Enzyme Commission (EC) classify enzymes mainly according to the following factors: (i) their catalytic action patterns against specified substrates (endo- or exo-), (ii) the type of linkages they hydrolyze (β-1,3), (iii) the substrate hydrolyzed (β-glucan/β-glucanase) and (iv) the type of reaction catalyzed (hydrolase) [70,82].

According to traditional EC nomenclature, the enzymes which catalyze the hydrolysis of β-1,3-D-glycosidic bonds are subdivided into the following classes: (i) EC 3.2.1.58: exo-β-1,3-glucanases, which cleave D-glucose from the nonreducing end of glucan molecule, (ii) EC 3.2.1.39: endo-β-1,3-glucanases, which require at least two β-1,3 bound glucose residues adjacent to the digested bond and (iii) EC 3.2.1.6: less specific endo-1,3(4)-β-glucanases, which are able to hydrolyze both β-1,3 and β-1,4 bonds, as long as the glucose residue whose reducing group is involved in the linkage to be hydrolyzed is itself substituted at C-3 [3,25].

Many glycoside hydrolases exhibit broad substrate specificity, making it difficult to use a traditional classification. The accumulation of data about the structure of glycoside hydrolases has led to the elaboration of a new system for classification of these enzymes based on the homology of amino acid sequences, their 3D structures and mechanisms of action. The new classification integrated glycoside hydrolases and their homologs into glycoside hydrolase (GH) families (Table 1). At the present time, there are over 170 different families present in the online CAZy database [83]. A glycoside hydrolase is included in a GH family if its primary sequence is homologous to the sequence of at least one already characterized member. Sixty-nine GH families have been grouped into 18 clans due to their common evolutionary origin, 3D structure similarity, conservative arrangement of catalytic residues and similar mechanism of glycosidic bond hydrolysis. The majority of β-1,3-glucanases are distributed between five GH families (5, 16, 17, 55 and 81). Nearly all characterized exo-β-1,3-glucanases (EC 3.2.1.58) are of fungal origin and have been assigned to GH families 5 and 55. In turn, endo-acting laminarinases (EC 3.2.1.39) seem to have a wider structural variety, having members in a wide range of GH families. Nonetheless, all endo-β-1,3-glucanases produced by plants can be assigned to the glycoside hydrolase family 17, whereas most of the bacterial ones, in addition to those from marine mollusks, belong to GH16. Fungal endo-β-1,3-glucanases, on the other hand, are more widely distributed across families but mostly belong to GH81. Classified endo-β-1,3(4)-glucanases (EC 3.2.1.6) are either produced by bacteria or fungi and most have been assigned to GH family 16 (Table 1) [71,76,84,85,86,87].

### 3.2. Primary Structure of β-1,3-Glucanases

Although some features remain constant, there is a significant degree of diversity between the primary structures of β-1,3-glucanases. As expected, the most conserved parts in β-1,3-glucanase amino acid sequences correspond to the active center and substrate-binding sites. The catalytic center of most endo-β-1,3-glucanases is characterized by the sequence GEIDIXE (with X being a hydrophobic amino acid residue). This sequence contains two important glutamic acid residues that function as the catalytic dyad, with the first assuming the role of catalytic nucleophile and the second that of the proton donor [3,25]. The substrate-binding site is a conserved tryptophan-rich sequence (WPAIWML). These conserved tryptophan residues were shown to promote substrate binding and positioning inside the active center cavity, by means of aromatic stacking interactions with the pyranose rings of glucose residues [71,74].

The importance of other residues for the catalytic activity of endo-β-1,3-glucanases was also shown. Michel et al. (2001) suggest that the conserved aspartic acid of the active site GEIDIXE cooperates with histidine residues in proton trafficking during the deglycosylation step [88]. In endo-β-1,3-glucanases of mollusks, two conserved cysteine residues seem to form disulfide bonds, which are determinant for the thermostability of the molecules [25].

### 3.3. 3D Structure of β-1,3-Glucanases

**Figure 3:**
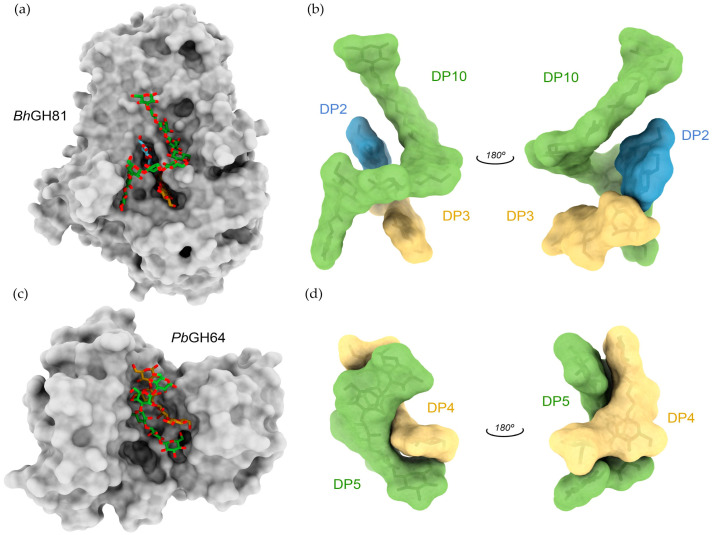
Tridimensional structure of *Bh*GH81 and *Pb*GH64 in complex with laminarin oligosaccharides. Gray-colored Van der Waals surface of (**a**) *Bh*GH81 and (**c**) *Pb*GH64, with the laminarin oligosaccharides in stick representation bound to the active site, highlighting the β-glucan quaternary structure recognition feature of both enzymes. Panels (**c**,**d**) show a detailed view of the pseudo-helical structures formed by the bound oligosaccharides. Each oligosaccharide strand is represented in a different color and identified with their degree of polymerization. In (**b**), DP2 and DP3 are likely to be the product of hydrolysis of *Bh*GH81 on a DP5 laminarin chain, as this is the structure of an active wild-type enzyme. PDB codes: 5t4g (*Bh*GH81), 5h9y (*Pb*GH64).

Currently, there are 32 experimentally determined 3D structures of β-1,3-glucanases [83]. Although the members of a given GH family can have different primary sequences and substrate specificity, they typically have the same type of fold and the same mechanism of hydrolysis (retaining or inverting). GH16 family members adopt a β-jelly-roll fold, GH5 and 17 families adopt a (β/α)8 (TIM-barrel) fold, GH55s have a β-helix conformation, and GH81s have a (α/α)_6_ barrel structure [83]. For example, the overall structure of a GH16 endoglucanase from *Pyrococcus furiosus* (pfLamA) exhibited a classical sandwich-like β-jelly-roll fold formed by two antiparallel sheets facing each other, with seven and eight strands, respectively. This structure is similar to that of BglF, an homologous GH16 endo-β-1,3-glucanase from *Nocardiopsis* sp. Surprisingly, despite the high degree of homology, BglF shows a preference towards MLG hydrolysis, whereas *pf*LamA is more active on β-1,3-glucans [89]. The crystal structure of a GH16 family β-1,3-glucanase from *Streptomyces sioyaensis* showed a sandwich-like β-jelly-roll fold with two disulfide bonds. Cysteine mutants decreased their optimal temperature, which indicates that the disulfide bonds are important to maintain thermostability [90]. In turn, the structures of plant GH17 endo-β-1,3-glucanases reveal a characteristic TIM-barrel fold defined by eight parallel strands in the interior of the protein surrounded by a ring of helices [91]. Despite β-1,3-glucanases of families GH16 and GH17 not having significant sequence similarity and unrelated three-dimensional structures, they show similar substrate specificity and activity, posing as an excellent example of the functional convergent evolution phenomenon [92]. Typically, substrate specificity is dictated by the active center topology, which is defined as a “pocket” or “crater” for exo-type β-1,3-glucanases, and as a “cleft”, “groove” or “tunnel” for endo-type enzymes. In the case of endo-β-1,3-glucanases, the tertiary and quaternary structures of β-1,3-glucans seem to play a particularly important role in substrate recognition. Two great examples of quaternary structure recognition are the GH81 β-1,3-glucanase from *Bacillus halodurans* (*Bh*GH81) and the GH64 β-1,3-glucanase from *Paenibacillus barengoltzii* (*Pb*GH64) [35,36]. *Bh*GH81 is an inverting endo-β-1,3-glucanase whose catalytic module adopts an (α/α)_6_ barrel structure. X-ray data from *Bh*GH81 crystals soaked with laminarin revealed three intertwined β-glucan chains bound to the catalytic site, resembling the triple-helix structure proposed for β-glucans (Figure 3a,b). The authors propose that the ability of *Bh*GH81 to recognize and extensively interact with the large quaternary structure of β-glucans favors a processive mechanism, in which the enzyme is not required to completely release the substrate after bond hydrolysis, but rather slides along the triple-helical structure and continues its catalytic action [36]. Much like *Bh*GH81, *Pb*GH64 is also an inverting endo-β-1,3-glucanase, but with a different tridimensional fold. Nonetheless, the clamp-like binding groove formed between a barrel domain and a mixed α/β domain possesses sufficient area to also be able to bind triple-helical β-1,3-glucan. Co-crystallization of *Pb*GH64 with laminarihexaose revealed two intertwined oligosaccharides in the catalytic groove, which were used to fit the 3D structure of curdlan, confirming *Pb*GH64’s ability to recognize its quaternary structure (Figure 3c,d) [35].

## 4. Other β-Glucan Binding Proteins

Most biological roles and applications of β-glucans are heavily dependent on their interaction with a wide variety of proteins. These β-glucan binding proteins range from substrate attachment-mediating non-catalytic modules present in β-1,3-glucanases, to cell surface receptors that modulate immune responses towards certain pathogens. They are widely distributed in nature and most seem to share a common feature: ligand configuration is crucial for recognition.

Below we briefly review some of the main β-glucan interacting proteins found in nature, while focusing on their biological role and the mechanisms supporting ligand recognition.

### 4.1. Carbohydrate Binding Modules

CAZymes are often multi-modular enzymes that possess one or even multiple ancillary modules, in addition to the catalytic one. The most common type of accessory modules are the carbohydrate binding modules (CBMs), which are non-catalytic motifs with the ability to recognize carbohydrates, tethering the enzyme to its cognate substrate [93]. The proximity effect created by the association of an enzyme with a particular substrate through its CBM results in enhanced enzymatic activity of the catalytic module [94]. Like CAZymes, CBMs have also been classified into families according to primary sequence homology. Currently, there are nearly 90 different CBM families, a number that has been steadily growing. Furthermore, CBMs fall into three distinct categories: type A CBMs, which interact with crystalline polysaccharides, mostly cellulose; type B CBMs, which bind to internal regions of single glycan chains; and type C CBMs, which target small saccharides at the ends of complex polysaccharides [95]. Much like CAZymes, the specificities of CBMs cover a variety of different polysaccharide ligands, including β-1,3-glucans and β-1,3-1,4 mixed linked glucans, which are recognized as CBMs from families 4, 6, 13, 32, 39, 43, 53, 54 and 56 [96,97,98,99,100,101]. Although CBMs from families 65, 72, 76, 79, 80, 81 and 85 have also shown affinity towards MLGs, no β-1,3-glucan binding has been reported. The fact that these can also bind to cellulose suggests that they recognize the 1,4 linked portions of the MLGs rather than the 1,3 linked regions [102,103].

**Figure 4:**
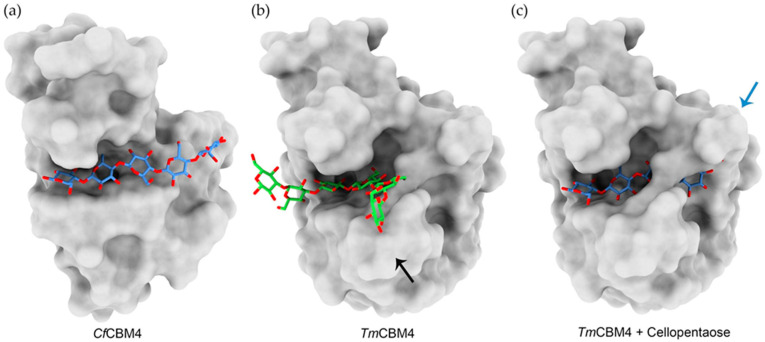
Substrate specificities of different CBM4 are dictated by active site conformation. Gray-colored Van der Waals surfaces of (**a**) *Cf*CBM4 bound to cellopentaose (blue) and of (**b**) *Tm*CBM4 bound to laminarihexaose (green). Both ligands are shown in stick representation. *Tm*CBM4 shows a more “U-shaped” binding site as a result of two loop extensions (black and blue arrows). An extra loop (black arrow) on *Tm*CBM4 creates an extra binding surface that interacts with the non-reducing end of the laminarin oligosaccharide. Panel (**c**) was obtained by superposing the structures of *Cf*CBM4 and *Tm*CBM4, and shows how the second extra loop on *Tm*CBM4 (blue arrow) does not allow the more linear cellulose oligosaccharide to fit into the binding site. PDB codes: 1gu3 (*Cf*CBM4), 1gui (*Tm*CBM4).

The binding specificities of CBM modules are usually related to the reported activity of their parent enzymes, which is why β-1,3-glucanases often possess one or more β-1,3-glucan-binding CBMs. Much like with endo-β-1,3-glucanases, structural analyses of β-1,3-glucan–specific CBMs have shown that one of the main drivers of ligand preference is the recognition of the β-1,3-glucan helical structure. A notable example is the family 4 CBM for *Thermotoga maritima* (*Tm*CBM4), a member of a CBM family with various ligand specificities. Its tridimensional structure was addressed by molecular replacement using the CBM4 from *Cellulomonas fimi* (*Cf*CBM4), which implies high homology between both CBMs. In fact, the overall structural folds of both CBMs are very similar and the main ligand binding residues are conserved between the two. However, these CBMs recognize quite different polysaccharides: *Cf*CBM4 is cellulose specific, whereas *Tm*CBM4 only recognizes β-1,3-glucans [96]. The reason for such similar proteins within the same CBM family having distinct specificities comes down to differences in the shape of the polysaccharide binding groove, which is more “U-shaped” in *Tm*CBM4 (Figure 4a,b), perfectly fitting the natural helical conformation of β-1,3-glucans. Moreover, an additional loop prevents any linear polysaccharide, such as cellulose, from fitting into *Tm*CBM4’s active site, further contributing to its β-1,3-glucan specificity (Figure 4c) [96].

More recently, studies on family 56 CBMs found that these modules are capable of recognizing both soluble and insoluble β-1,3-glucans, but do not bind β-1,3-glucooligosaccharides. Analysis of its tridimensional structure allowed the identification of features consistent with binding of the triple-helical quaternary structure of β-1,3-glucopolysaccharides, explaining the lack of affinity for the linear β-1,3-glucooligosaccharides. Furthermore, ITC analysis showed a relatively low number of CBM56 binding sites in laminarin isolated from *Laminaria digitata*, which is consistent with the fact that only ~5% of laminarin from this source forms triple helices [84].

### 4.2. Dectin-1

**Figure 5:**
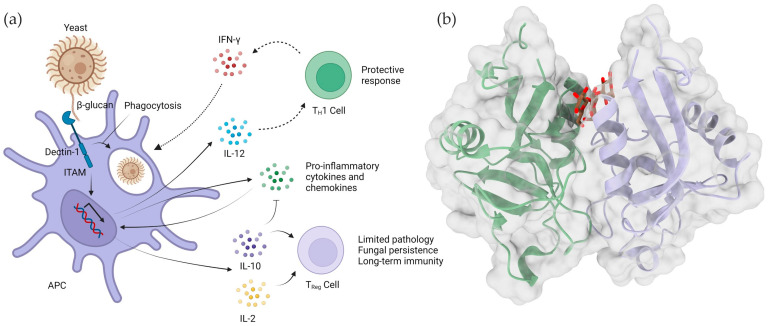
Dectin-1 activation by fungal β-glucans. (**a**) Dectin-1-mediated anti-fungal immunity via β-glucan binding is a consequence of several cellular responses, including fungal uptake and killing [111,112], and the production of pro-inflammatory cytokines and chemokines that lead to immune cell recruitment and activation [113,114]. Dectin-1-mediated recognition also stimulates the production of interleukin-12 (IL-12), which elicits a protective T_helper_ 1 (T_h_1)-cell response with the production of interferon-γ (IFN-γ), thereby activating fungal killing by the phagocytes [113,115]. In dendritic cells, Dectin-1 activation can also induce the production of IL-10 and IL-2 [115], leading to the development of regulatory T cells, which prevent pathological inflammation and promote long-term immunity [116]. IL-10 also inhibits the production of pro-inflammatory cytokines and chemokines (based on a figure by Brown, G. 2006 [117]). Panel (**b**) shows a cartoon representation of the 3D structure of dimer of two Dectin-1 lectin modules (green and blue), with a trapped β-glucan oligosaccharide between the two units, in brown (PDB code: 2cl8).

Dectin-1 is a 28 KDa type II transmembrane protein that can bind to β-1,3 glucans, with or without β-1,6 branching. The name Dectin-1 comes from “Dendritic-cell-associated C-type lectin-1”, due to it being initially described as a dendritic cell receptor. However, Dectin-1 was later discovered to be implicated in the elimination of fungal pathogens by macrophages, neutrophils and dendritic cells, and to be expressed on most innate immune system cells [104,105]. Dectin-1 is composed of an extracellular lectin domain capable of β-glucan recognition, a transmembrane region and a cytosolic domain with an immunoreceptor tyrosine-based activation motif (ITAM), which mediates a ligand-induced activation of a signaling response, resulting in the formation of a vast array of immune modulators (Figure 5) [106].

Dectin-1’s extracellular lectin domain is responsible for carbohydrate recognition, binding to β-1,3 glucans in a chain length-dependent fashion, with a seemingly higher affinity for ligands with larger DP [107]. Although the mechanism of the chain length-dependent interaction is unclear, increased β-glucan chain length correlates with increased secondary structure formation. As such, it is likely that the conformation of Dectin-1’s ligand-binding site is capable of more easily accommodating those quaternary helical structures, justifying its preference for longer polysaccharides [108]. In addition, 1,6-branching also seems to affect binding to Dectin-1, with branched polysaccharides having increased affinity in comparison to linear ones [109].

Although Dectin-1’s best-described function is as a fungal and yeast pathogen recognition receptor, some studies suggest that it may have a broader function in pathogen recognition, including a role in directing macrophage response to mycobacterial infections [106]. Furthermore, other functional aspects have also been described for Dectin-1, such as binding to the conserved core domain of annexins in apoptotic cells to induce immune tolerance and the recognition of N-glycans decorating IgG antibodies, a feature whose biological importance is still under investigation [110]. Therefore, Dectin-1 seems to be a uniquely versatile lectin-like molecule with multiple roles in immune modulation [49].

### 4.3. Complement Receptor 3

Complement receptor 3 (CR3) is a receptor belonging to the β_2_-integrin family, consisting of a heterodimer between the α_M_ (CD11b) and β_2_ (CD18) subunits. It is one of the most versatile receptors expressed by NK-cells, neutrophils and lymphocytes, mediating adhesion, chemotaxis and phagocytosis, both in a complement-dependent and -independent manner [118,119].

CR3’s CD11b subunit possesses a lectin-like site, which is capable of recognizing β-glucans. Interestingly, this lectin domain is involved in a double recognition mechanism that triggers immune cells, leading to a cytotoxic response. β-glucans first bind with high affinity to the lectin site and the overlapping I-domain of CD11b, changing CR3’s conformation, priming it for recognition of complement component iC3b [120,121]. Circulating immune cells with primed CR3 will then bind to iC3b-opsonized cells, which can be of any type, including cancer cells tagged with monoclonal antibody (mAb) and coated with iC3b, leading to their elimination through cytotoxicity [122]. Together with Dectin 1’s activation, these are currently the two best described mechanisms of β-glucan-mediated immunomodulation in vertebrates.

### 4.4. β-glucan Binding Proteins in Invertebrates

Unlike vertebrates, insects and crustaceans lack the ability to develop acquired immunity, relying on their innate immune system for defense against external pathogens. Many of the pathways involved in arthropod immunity are triggered upon the binding of proteins, described as β-(1-3)-glucan recognition proteins (βGRPs), with β-glucans expressed on the surface of pathogens. These βGRPs comprise one of the best characterized families of pattern recognition receptors in invertebrates and are widely distributed among species [123]. The βGRP/Gram-negative binding protein 3 (GNBP3) is one of the best studied examples of βGRPs involved in insect immunity. It uses its β-glucan binding capabilities to detect fungal invasions, triggering a proteolytic cascade that eventually leads to the activation of the Toll pathway and subsequent production of antimicrobial peptides. βGRP/GNBP3 β-glucan binding occurs via its immunoglobulin-like N-terminal domain (N-GNBP3), which is highly conserved among insect species [101,124,125,126]. The molecular mechanism of the interaction is still controversial, as different studies suggest distinct β-glucan binding sites. Although NMR titration experiments and mutational analysis of the silkworm’s (*Bombyx mori*) GNBP3 suggested that β-glucan binds to the concave surface, its structure in complex with laminarihexaose reveals the ligand bound on the convex surface [101,125]. Nonetheless, even though GNBP3 has a high degree of conservation across species, there is evidence suggesting the existence of two groups with distinct binding specificities. A recent study on βGRP/GNBP3s from four different insect species (*Bombyx mori*, *Plodia interpunctella*, *Tribolium castaneum* and *Tenebrio molita*), using solid phase ELISA assays, revealed that one group (comprising *Bombyx mori* and *Plodia interpunctella*) preferentially binds to triple-helical β-glucans, whereas the other (*Tribolium castaneum* and *Tenebrio molita*) is not capable of recognizing the quaternary structures, but is able to bind alkaline-treated β-glucans with a partially open structure [127]. Although these results suggest that ligand conformation is crucial for recognition by βGRP/GNBP3 and that the preferred configuration is different for an individual βGRP/GNBP3, the structural mechanism behind preference remains to be determined.

Another well-known β-glucan binding protein is the Factor G from the horseshoe crab. This hemolymph coagulation factor consists of an heterodimer with an α-subunit (72 kDa) and a β-subunit (37 kDa), which are autocatalytically cleaved into the active form upon binding to β-glucan [128]. The α-subunit is responsible for the recognition of β-glucan, whereas the β-subunit is a serine protease that becomes activated when factor G binds to β-glucan. Two distinct β-glucan recognition sites are present in factor G and, although both can independently bind to ligands with a wide range of DP, proper activation of the clotting cascade requires interaction with long chain β-glucans through both sites [129]. Therefore, this protein functions as a biosensor for the longer (1,3)-β-glucan present on pathogenic fungi, which is why it is used as a diagnostic reagent for the detection of fungal infections in humans [130].

As in the case of most β-glucan interacting proteins described above, the natural triple-helical structure of β-glucan seems to be a key factor for the interaction, with the activation of Factor G increasing over 100-fold after alkaline treatment of β-glucan [131].

## 5. Applications of β-Glucans

β-glucans are biologically active biopolymers that can interact with several protein receptors, present both in micro- and macro-organisms, eliciting a wide spectrum of biological responses. These responses often result in important bioactivities including immunomodulating, anticancer, antioxidant, cholesterol-lowering and prebiotic effects [5,7,8,9,132]. Naturally, it is not surprising that numerous studies have tried to harness the potential therapeutic properties of β-glucans, which can be generally divided into immunomodulatory or metabolic properties [10,11,12,13,14,15,133,134].

Due to their physicochemical properties, β-glucans also have a wide range of potential applications in several industries, such as a thickening and gelification agent in the food industry, and as a soothing, moisturizing and skin regenerating agent in the cosmetic and personal care industry [19,20]. They have also inspired several ingenious biotechnological and biomedical solutions, ranging from hydrogel scaffolds for 3D cell cultures to drug delivering nanoparticles [135,136].

Here, we summarize some important biomedical, biotechnological and industrial applications of β-glucans.

### 5.1. Immunomodulating Activity

The anecdotal recognition of the immune-modulating properties of β-glucans as the main active component of mushrooms has long been well established. However, its effects in promoting immune response and boosting resistance to infections have only been scientifically proven in recent decades [137,138]. The exact mechanisms by which β-glucans activate the immune system are complex, depending on many factors that have not yet been fully revealed [14]. Unfortunately, it does not help that many of the studies fail to mention the exact origin of the β-glucans used, although it seems that most immunomodulatory trials are performed with fungal β-1,3-glucans.

Despite that, β-glucans’ ability to modulate the immune system has been associated with its interaction with several PRRs present on the surface of various immune cells, including monocytes, macrophages, neutrophils and natural killer (NK) cells. Since β-glucans are not produced by mammalian systems, these polysaccharides are recognized by the host’s innate immune response as foreign, acting as biological response modifiers [139]. To date, several different β-glucan PRRs have been described, including complement receptor 3 (CR3), scavenger receptors, lactosylceramide and Dectin-1 [140,141,142,143]. The interaction between β-glucans and PRRs triggers a signal transduction that, depending on the immune cell type, boosts a specific immune response [137].

For instance, research in this field revealed that neutrophil modulation by β-glucans is mainly CR3 dependent, whereas Dectin-1 is the most predominant β-glucan receptor on macrophages [122,144]. By binding to the lectin site of the CR3 on phagocytes and NK cells, β-glucans activate this receptor, promoting cytotoxicity against iC3b-opsonized target cells [145]. In turn, the interaction of Dectin-1 on macrophages with β-glucans triggers a downstream signaling pathway, activating phagocytosis, ROS generation, microbial killing and cytokine production [142,146].

Initially, it was thought that β-glucans’ immunomodulating properties were solely associated with the stimulation of T cell-independent immune responses [147]. However, there is increasing evidence of the influence of β-glucan in T cell differentiation. Because they consist of polysaccharides having both positive and negative charges, β-glucans can activate CD4^+^ T cells through the MHC-II endocytic pathway [148]. After being processed into smaller molecular weight carbohydrates, they can bind to MHC-II inside the antigen-presenting cells and are subsequently presented to T_helper_ cells. In addition, β-glucans immune-related responses lead to the polarization of T cells. For example, dendritic cells activated by the bacterial β-glucan curdlan have been shown to promote T_h_1, T_h_17, and cytotoxic T lymphocyte priming and differentiation [149,150], and T_reg_ conversion into IL-17 producing T cells [151] via the Dectin-1–dependent pathway. Moreover, the yeast zymosan β-glucan seems to stimulate regulatory antigen-presenting cells, leading to T_reg_ differentiation [152,153].

Notably, recent studies have demonstrated the ability of β-glucan to prime innate immune cells in a robust manner, a concept defined as trained innate immunity, in which a secondary immune stimulus involves a heightened activation of innate immune cells [154,155]. Evidence has demonstrated a stronger innate response from monocytes and macrophages after β-glucan exposure, with improved antimicrobial and inflammatory properties consequent on Dectin-1/toll-like receptor (TLR) activation [156]. However, trained innate immune cells show less specificity and duration of memory, when compared to the classical acquired immunity promoted by antigen-specific responses.

These regulatory effects on immune response are considered one of the most promising biological functions of β-glucans, making these compounds appealing immunomodulating agents for therapeutic interventions.

#### 5.1.1. Anticancer Activity

The first evidence of the antitumor activity of β-glucans was published almost 40 years ago [157]. Since then, several studies have proven the potent anticancer activity of β-glucans against various types of malignancies, including lung, breast and gastrointestinal cancers [158,159,160,161,162,163,164,165,166]. This promising clinical efficacy culminated in the approval of lentinan as a biological response modifier for cancer therapy in Japan, and its clinical use for over 30 years [167].

Lentinan has been considered an adjunctive therapy to radio/chemotherapy for patients diagnosed with solid tumors, improving response rate, one-year survival, performance status, quality of life and radio/chemotherapy toxicity [168]. A clinical trial evaluated the intravenous administration of lentinan extract in combination with two chemotherapeutic compounds (5-FU and Tegafur) in patients with advanced or recurrent stomach, colorectal and breast cancers. Lentinan treatment in combination with chemotherapeutic agents promoted life span prolongation and was associated with the improvement in host immune response [169].

The anticancer effects of β-glucan-based therapies were also clinically associated with immune function regulation. Schizophyllan was used in the treatment of advanced cervical cancer, improving the function of T_helper_ lymphocytes and enhancing the IL-2/IL-2R system [163]. Schizophyllan combined with chemotherapy enhanced the long-term survival rate of patients with ovarian cancer [170]. Further clinical trials evaluated β-glucans therapeutic efficacy in patients with cancer, and as an adjunctive therapy in patients receiving chemotherapy to limit suppression of hematopoiesis. Data gathered from a clinical trial demonstrated that β-glucans can be safely administered to patients with advanced malignancies receiving chemotherapy and that this adjunctive therapy may ameliorate adverse effects of chemotherapy on blood counts [171]. Furthermore, a significant reduction in chemotherapy-associated side effects such as loss of appetite, alopecia, emotional instability and general weakness was reported in β-glucan supplemented patients, leading to a general improvement in quality of life [172,173,174,175,176].

β-glucans antitumor responses appear to vary depending on their origin, structure and composition [177]. For example, zymosan, a yeast-derived mixture of β-1,3-glucan and protein complexes, elicits immune responses by enhancing the number and function of macrophages while activating the complement system [178]. Lentinan triggers antitumor immune responses by improving the lymphokine-activated killer cell activity and NK cell activity [179]. A yeast-derived particulate β-glucan promotes antitumor immune responses by triggering pro-inflammatory cytokine secretion and stimulating innate immune effector cell activation [180]. Overall, these findings support the notion that β-glucan-based agents mediate antitumor immune responses through different mechanisms.

**Figure 6:**
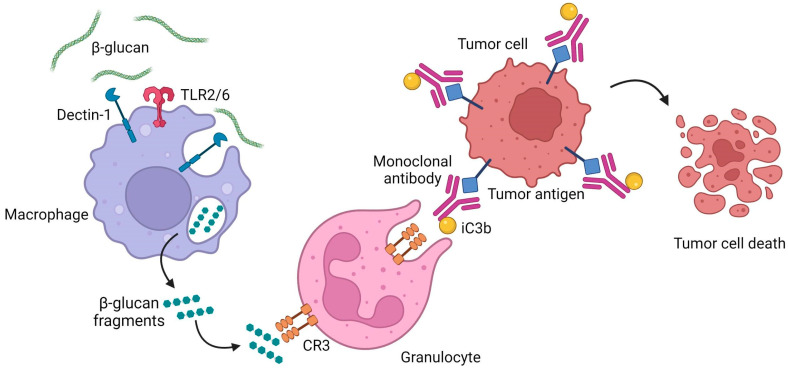
Complement-dependent antitumor cytotoxicity mediated by β-glucans. Due to the lack of β-glucans, tumor cells are not able to induce dual binding to leukocytes, promoting complement-dependent cytotoxicity. However, the introduction of exogenous β-glucans can generate dual-binding of leukocytes to iC3b-positive tumor cells. β-glucans are captured and internalized by the macrophages via the Dectin-1 receptor with or without TLR-2/6. The large β-glucan molecules are fragmented into smaller sized β-glucan fragments within the macrophages and are carried to the marrow and endothelial reticular system and subsequently released. These small β-glucan fragments are eventually recognized by the circulating granulocytes, monocytes or macrophages via the complement receptor (CR)-3, priming leukocytes against tumor cells opsonized with iC3b by monoclonal antibodies.

One of the most interesting β-glucan-mediated antitumor immune responses is the possibility of using β-glucans to trigger complement-dependent antitumor cytotoxicity [177]. Complement is a key component of the innate immunity against β-glucan presenting microorganisms. Because these molecules are not expressed by tumor cells, tumor cells are not capable of triggering CR3-dependent cellular cytotoxicity [181]. As such, by activating the CR3 receptor on innate cells, such as macrophages, dendritic cells, natural killer cells and neutrophils, β-glucans have the ability to prime leukocytes to mediate a specific cytotoxic immune response against previously iC3b-coated tumor cells by circulating anti-tumor antibodies. Therefore, β-glucans may function as a potent adjuvant for cancer mAb therapy to elicit a novel granulocyte and tissue macrophage-mediated tumor-killing mechanism that is not activated by mAb therapy alone [182] (Figure 6).

Several murine syngeneic tumors [182,183] and human carcinoma xenograft models [184,185,186] have attested to the significant therapeutic efficacy of combined β-glucan and anti-tumor mAbs therapy. Moreover, there are multiple clinical trials for β-glucan-based cancer immunotherapies, many of which combine β-glucans with mAbs (Table 2) [187]. A phase I clinical trial enrolling 20 patients with chronic lymphocytic leukemia investigated the combined treatment using mAbs with PGG glucan to study the hypothesis that β-glucans would augment the cytotoxic activity of the innate immune system when administered in association with mAbs. Data showed that treatment was well-tolerated by patients, which justified a further phase II study. Interestingly, while monotherapy with rituximab and alemtuzumab rarely achieved a complete response or a sustained response, its combination with β-glucans resulted in a high complete response rate. Although this study gave hope that the addition of β-glucans may improve the response to mAb therapies, due to the study’s small size, the preliminary data did not allow conclusions to be drawn [188]. A similar clinical trial was conducted based on the same scientific hypothesis using the same PGG glucan in combination with carboplatin/paclitaxel chemotherapy plus an EGFR-targeted antibody (cetuximab) in untreated stage IIIB/IV non-small cell lung cancer patients. Results demonstrated that the addition of β-glucans was well tolerated and significantly improved objective response rate in enrolled patients [189].

This concept has been also investigated using a combination therapy with β-glucans and immune checkpoint inhibitors, and has obtained encouraging results in preclinical studies. The combination treatment with β-glucans and an anti-PD-1 antibody elicited a coordinated immune response, which translated to a promising antitumor response in a syngeneic cancer murine model [190]. The benefit of β-glucans has also been evaluated in association with pembrolizumab in various phase II studies in cancer patients. Recently, a clinical trial studied the effect of combining PGG with pembrolizumab in subjects with chemotherapy-resistant metastatic triple negative breast carcinoma [191]. The combination of Imprime (a soluble yeast derived β-glucan) and this anti-PD1 antibody provided promising response rates and overall survival. Moreover, biopsy analyses consistently showed activation of both myeloid and T cells with extensive infiltration into tumor tissue. By acting as an immune-modulator of the innate and adaptive response, β-glucans have the potential to alter the tumor microenvironment towards an immunostimulatory phenotype, stimulating and improving clinical response to immune checkpoint inhibitors [192].

Another advantage of β-glucans in cancer immunotherapy is their synergistic action with cancer vaccines. By acting as potent adjuvants for tumor vaccines, β-glucans can elicit potent cytotoxic T cell responses along with humoral responses. The combination of a bivalent ganglioside vaccine with β-glucan was investigated in a phase I clinical trial for high-risk pediatric neuroblastoma in second or later remission. Data showed promising serological and minimal residual disease responses and that the vaccine/β-glucan treatment was well tolerated [193].

More recently, the promising immune modulatory effect of β-glucans has encouraged the generation of glucan-based multifunctional nanomedicine systems for targeted delivery of glucan in combination with therapeutic drugs to tumor cells. β-glucans were incorporated as carriers in a novel targeted delivery system loaded with doxorubicin conjugated to trastuzumab antibody for the treatment of HER2^+^ breast xenotransplant tumors in mice. These β-1,3-glucan-doxorubicin-targeted nanoparticles demonstrated superior tumor inhibition compared to the control groups [194].

Hence, considering its proven safety and low toxicity profile, along with the success of emerging combinatorial approaches for cancer treatment involving these molecules, β-glucans have the potential to revolutionize future strategies in immuno-oncology.

**Table 2:** Ongoing and completed clinical trials involving β-glucans for cancer treatment, according to the clinicaltrials.gov database (as of 25 February 2022) [195].

Molecule	Cancer Type	Status	Identifier
Yeast derived β-glucan + glutamine + immunoglobulin	Metastatic cancers	Phase II/III	NCT04710290
Yeast derived Soluble Beta-Glucan	Advanced solid tumors	Phase I	NCT01910597
Soluble β-1,3-1,6-glucan + standard antibody and chemotherapy	Breast cancer	Phase I/II	NCT00533364
Soluble β-glucan + rituximab + COP/CHOP	Non-Hodgkin’s lymphoma	Phase I	NCT00533728
Particulate β-glucan	Oral squamous cell carcinoma	Not Applicable	NCT04387682
β-glucan MM-10-001	Locally advanced or metastatic non-small cell lung cancer	Phase I	NCT00857025
β-glucan (Imucell WGP)	Non-small cell lung cancer	Not Applicable	NCT00682032
β-glucan + pembrolizumab	Melanoma stage III/IV	Not Applicable	NCT04513028
β-glucan + monoclonal antibody 3F8	Neuroblastoma	Phase I	NCT00037011
β -glucan + monoclonal antibody 3F8	Neuroblastoma	Phase I	NCT00492167
β-glucan + isotretinoin + sargramostim + monoclonal antibody 3F8	Neuroblastoma	Phase II	NCT00089258
β-glucan + granulocyte-macrophage colony stimulating factor + bivalent vaccine + adjuvant OPT-821	Neuroblastoma	Phase II	NCT04936529
β-glucan + bivalent vaccine + adjuvant OPT-821	Neuroblastoma	Phase I/II	NCT00911560
Imprime PGG β-glucan + rituximab + alemtuzumab	Chronic lymphocytic leukemia	Phase I/II	NCT01269385
Imprime PGG β-glucan + pembrolizumab	Malignant Neoplasm of Breast	Phase II	NCT05159778
Imprime PGG β-glucan + pembrolizumab	Advanced melanoma Triple-negative breast cancer	Phase II	NCT02981303
Imprime PGG C + rituximab	Relapsed/refractory indolent B cell non-Hodgkin lymphomas	Phase II	NCT02086175

#### 5.1.2. Anti-Infective, Anti-Inflammatory and Wound Healing Properties

β-Glucans have also shown interesting biological effects, such as anti-microbial, anti-inflammatory and wound healing properties. Several studies suggested that β-glucan supplementation improved mucosal innate immunity and reduced upper respiratory tract infection incidence and symptom severity in pediatric patients [196,197,198]. β-glucan supplementation may be also associated with defense against infection in adults and the elderly [199,200]. In particular, there is some evidence that adults suffering from allergies benefit from β-glucan supplementation to reduce symptom severity [201]. Very few studies have been performed on autoimmune diseases; however, an extract derived from *Agaricus blazei* was tested in inflammatory bowel diseases, with modest results on inflammatory cytokines or clinical symptoms [202,203]. Moreover, β-glucans boost wound repair by increasing the infiltration of macrophages, which promote tissue granulation, collagen deposition and skin re-epithelialization [204]. Human clinical studies provided evidence that β-glucan can accelerate healing of chronic wounds. The application of a 3% cream containing curdlan caused a 55% reduction in ulcers after 90 days of treatment [205]. Another clinical study demonstrated that the utilization of soluble yeast β-1,3-glucan allowed 59% of total ulcers to be healed compared to 37% of the control group by week 12 [206].

### 5.2. Metabolic Activity

Beyond their immunomodulatory effects, β-glucans, particularly MLGs, also present important metabolic and gastro-intestinal effects through the modulation of gut microbiota, alteration of lipid and glucose metabolism, and reduction in cholesterol. These beneficial effects led to a variety of studies regarding β-glucans as potential therapies for metabolic syndrome, obesity and diet regulation, and gastrointestinal conditions, such as irritable bowel, and to lower cardiovascular and diabetes risk [7]. Remarkably, evidence supporting the beneficial role of oat β-glucans led the US Food and Drug Administration (FDA) to approve the use of health claims linking oat products with the reduction in coronary heart disease risk [207].

β-glucans decrease the blood postprandial glycemic and insulin peak by creating a barrier in the small intestine that hinders glucose absorption [192]. Furthermore, there is increasing evidence of β-glucans’ influence on the activation of metabolic pathways through phosphatidylinositol 3-kinase (PI3K)/serine-threonine kinase (Akt), a key signaling pathway of the pathogenesis of diabetes [208]. A systematic review and meta-analysis conducted by Zurbau et al. (2021) confirmed that adding oat β-glucans to carbohydrate-containing meals reduces glycemic and insulinemic responses [209].

In turn, the cholesterol lowering effects of β-glucans are proposed to be mainly mediated by the gel-forming properties of β-glucans, which modulates bile acid and cholesterol metabolism. β-glucans attenuate the intestinal uptake of dietary cholesterol while preventing bile acid reabsorption that subsequently increases the demand for *de novo* synthesis of bile acids from cholesterol catabolism, which contributes to lowering the LDL fraction of circulating cholesterol [210]. However, more recently, the impact of β-glucans activity on gut microbiota modulation, particularly on those bacterial species that influence bile acid metabolism and production of short chain fatty acids, has been implicated in the regulation of cholesterol homeostasis [211]. Several randomized-controlled trials and subsequent meta-analyses reported a significant correlation between the consumption of oats or oat β-glucans and lower LDL cholesterol levels, while also reporting other enhanced markers of cardiovascular disease risk [212,213,214,215].

In addition, given their non-digestible and non-absorptive characteristics in the human gastrointestinal tract, β-glucans have been suggested to be dietary fibers with potential prebiotic properties [216]. β-glucans are reported to accelerate bowel transit, increase fecal bulk and frequency, and to positively influence gut microbiota regulation, preventing irritable bowel syndrome, diverticular diseases and colon cancer [7]. The growth of normal intestinal microbiota (*Lactobacilli* and *Bifidobacteria* species) are supported by β-glucans in vivo and in vitro models [217]. Even though strong evidence of the prebiotic effect of β-glucans in humans is still lacking, β-glucans prebiotic potential was confirmed in a clinical trial conducted by Mitsou et al. (2010). This work reported that daily intake of a barley β-glucan was well tolerated and had a bifidogenic effect, boosting the *Bifidobacteria* number to a detectable level [218].

Altogether, these results clearly demonstrate the potential that inclusion of β-glucans on diets may have in reducing the prevalence of cardiovascular disease, diabetes and gastrointestinal diseases, and its associated healthcare costs.

### 5.3. Industrial Applications of β-Glucans

Due to their unique physicochemical properties (solubility, viscosity and gelation), β-glucans have found potential applications in a wide range of industrial sectors, such as the agronomy, cosmetic, pharmaceutical and food industries. In the food industry, β-glucans are very popular thickening agents, but can also be used with many other purposes, such as: additives in the preparation of frozen sweet foods, improving the texture of cakes and the shape retention of ice creams [219,220]; to reduce the leaching out of soluble ingredients and promote softening in pasta noodles preparation [221]; as fat substitutes and gelling agents in the preparation of meat products [222,223]; and as components in the preparation of edible films for food packaging [224]. Another popular application for β-glucans is in the formulation of low-calorie foods [225,226]. For example, a high-fiber and low-calorie substitute for wheat flour was developed using β-glucan-enriched materials from the mushroom *Lentinus edodes* [227]. The authors suggested that this product can be used to bake cakes containing 1 g of β-glucan per serving with quality attributes akin to those of traditional formulations. Due to the potential metabolic effects of cereal β-glucans, they have also been widely explored as ingredients in the formulation of low-fat and cholesterol-lowering products, including salad dressings [228], milk [229], yogurt [230] and cheese [231]. In addition to the food industry, the cosmetics and personal care industry also make extensive use of β-glucans. Due to their soothing, moisturizing and anti-irritant properties, β-glucans have been a common ingredient in protective creams, ointments and powders, with oat β-glucan being particularly popular. For example, “Avenacare” is a liquid form of active oat β-glucan, naturally extracted from Swedish oats using a chemical-free technology [20]. There is also evidence showing that baker’s yeast β-glucan might promote keratinocyte growth, resulting in increased skin regeneration and protection against damage, and that low doses of laminarin from *L. digitata* provide antioxidant protection to the skin [232,233].

Due to the immunomodulating aspects of β-glucans, in addition to their ability to improve the gut microbiome and promote gastro-intestinal health, some studies suggest the use of β-glucans as feed ingredients in animal husbandry. There is evidence that they are capable of enhancing the natural immunity of animals, promoting productivity and decreasing the need for antibiotics [234,235,236]. The inclusion of yeast cell β-glucans in poultry diets was shown to increase the intestinal clearance of pathogens by protecting intestinal barriers, stimulating phagocytosis and suppressing pathogen invasion [237,238,239,240]. Supplementation of piglet diets with *Sacharomyces cerevisiae* β-1,3-glucans increased production performances and resulted in significant stimulation of phagocytosis by both peripheral blood monocytes and neutrophils, and improved responses to endotoxin challenge [241]. In aquaculture, where the vaccination process can be laborious and impractical, β-glucans have also been proposed as potential agents for prophylaxis and disease management [235]. In summary, with current efforts to eliminate the use of antibiotics and growth promoters in the livestock industry, the inclusion of β-glucans in feed may be a viable option, offering both natural growth stimulation and immune protection.

### 5.4. Biotechnological and Biomedical Applications of β-Glucans

Beyond their use as immunostimulants and metabolic enhancers, β-glucans have also had their properties explored in the development of new biotechnological solutions. Notably, there are a number of recent studies exploring the potential of β-glucans as a novel support for cell culture and as scaffolds to create 3D tissue constructs [135,242,243,244]. Martins et al. (2018) developed photopolymerizable microparticles from methacrylated laminarin, using a microfluidics device, which were incorporated with platelet lysates and further conjugated with an adhesive peptide [242]. The microparticles were seeded with mouse fibroblast L929 cells and the results showed enhanced cell adhesiveness to the laminarin microparticles and an increased proliferation rate after 11 days. Moreover, expanded cells provided the link for microparticle aggregation, resulting in self-assembled robust 3D structures. In another work, a 3D gel composite scaffold was designed using laminarin hydrogel and graphene foam. This composite scaffold exhibited a reinforced toughness and was utilized for 3D culture of human mesenchymal stem cells. The graphene foam supported cell attachment and cell spreading, while the laminarin hydrogel conjugated with an adhesive peptide regulated cell migration and formed an interconnected cellular network in the pores of the graphene foam [135]. In both studies, the authors suggest that their systems have the capacity to produce large tissue-engineered constructs, and thus to be applied in tissue engineering and regenerative medicine. The use of bulk hydrogels in cell culture poses severe limitations, such as oxygen, nutrient and metabolite diffusion. In an attempt to overcome these limitations, self-feeding laminarin-based hydrogels with immobilized β-glucanases were assembled and used to support the growth of 3D tumor and human stem cell cultures, while simultaneously providing a continuous source of glucose for the metabolic activity of cells, through laminarin degradation [245].

A number of recent studies have focused on the development of β-glucan-based drug delivery systems, either as hydrogels or for the encapsulation of therapeutically important biological agents, such as siRNA, peptides and DNA, which can lead to a new approach to immunization and cancer immunotherapy [136,246,247,248]. Notably, in an experimental glioblastoma stem cell therapy study, the utilization of β-1,3-glucan as an outer shell coating for paclitaxel-loaded chitosan nanoparticles was able to enhance chemotherapeutic potency, overcome systemic toxicities and improve drug bioavailability [249]. Another study suggested a multifunctional laminarin-based nanomedicine carrier biomaterial with dual pH/redox sensitivity that has the potential to be applied as a new drug delivery system for cancer therapy. The system achieved great results in delivering a photosensitizer for photodynamic therapy in breast cancer [250]. β-glucans are also a promising alternative to aluminum as vaccine adjuvants, due to their natural ability to promote immunogenicity [251]. Moreover, β-glucan can be prepared in a particulate form, consisting of spherical hollow particles of β-glucan, which can be loaded with different compounds, including peptide antigens, functioning as both as adjuvant and antigen carriers [252,253]. Another interesting study took advantage of the quaternary triple helix structure of β-glucans to develop schizophyllan–antigenic peptide complexes, by replacing one of the strands in the triple helix with a polyadenylic acid chain linked to a peptide. The complex was able to induce a strong antigen-specific cytotoxic T lymphocyte activity, resulting in an increased immunogenicity with a much lower peptide dose. This is due to the targeting effect of schizophyllan towards antigen-presenting cells through the interaction with β-glucan receptors, a strategy that can be used in the development of potent vaccines against infectious diseases and cancer [248]. In summary, there are myriad potential biotechnological and biomedical applications for β-glucans, but much of the research is still in its infancy. Nonetheless, it is evident that there is a growing interest in these subjects, which is reflected in the ever-increasing number of studies.

### 5.5. Applications of β-1,3-Glucanases

Much like their natural substrates, β-1,3-glucanases have found a number of applications in many industrial processes and serve as inspiration for several biotechnological solutions. They have been extensively studied for the use in the conversion of β-1,3-glucan containing lignocellulosic biomass, namely of algal origin, into fermentable sugars to be used in bioethanol production [80,254]. They offer a more ecological and energy -efficient alternative to classical methods such as chemical extraction at high temperatures, although the costs of producing the enzymes at a large scale are currently prohibitive [255]. Another popular application for β-glucanases is as a feed additive in animal production to improve growth and performance. Barley- and oat-based diets can significantly increase the viscosity of the digesta in monogastric animals, with poultry being particularly affected. This leads to slower gastrointestinal transit and decreased feed intake and nutrient absorption. In order to overcome these antinutritive effects, feed supplementation with β-glucanases has become a common option [31,256,257].

Given that β-1,3-glucans are major components of the fungal cell wall, β-1,3-glucanases possess an important antifungal activity. As such, they have attracted a great deal of attention as potential non-chemical biocontrol agents against fungal pathogens in crops [70,80]. Members of the genus *Trichoderma* are especially well known for their mycoparasitic activities against various phytopathogenic fungi. By combining β-1,3-glucanases from *Trichoderma harzianum* with chitinases, it was possible to achieve a potent antifungal activity against the phytopathogenic fungus *Botrytis cinera* or “grey mold”, which is responsible for serious economic losses to both field- and greenhouse-grown crops [258]. Because β-1,3-glucanases have no cytotoxic effects towards animal cells, their antifungal activity may also pose a potential and safe solution for the treatment of pathogenic fungal infections, particularly those caused by *Candida albicans* [34].

In brewing, the increased viscosity caused by barley β-glucans can cause a number of problems, notably reduced rates of wort separation and beer filtration, and also the formation of hazes, gels and precipitates. As such, β-1,3-glucanases are extensively used in brewing to avoid the accumulation of barley β-glucans, decreasing wort viscosity, improving filtration and promoting the elimination of unwanted beer turbidity. They have also been shown to be able to speed up germination during the malting process without compromising beer quality and organoleptic features [30,259].

**Figure 7:**
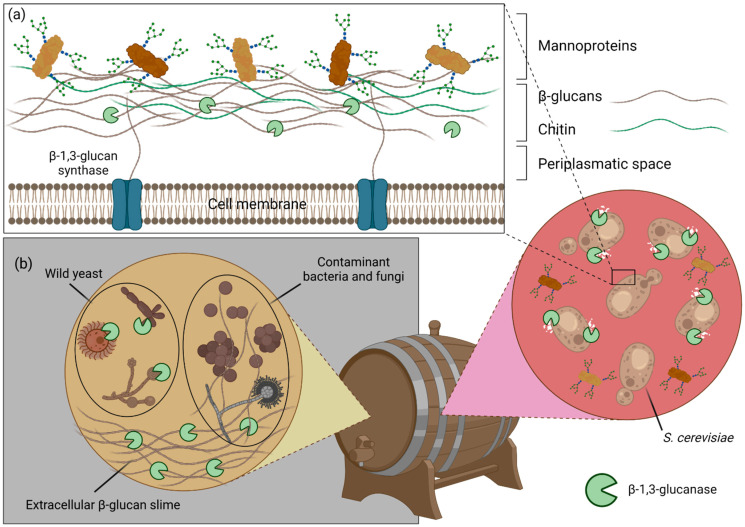
Applications of β-1,3-glucanases in the winemaking process. (**a**) The starter yeasts, usually *S. cerevisiae*, possess several components in their cell wall, most importantly mannoproteins that, when released into the wine, improve several organoleptic and technological properties of the final product. By hydrolyzing yeast cell wall β-glucans, β-1,3-glucanases promote the release of those mannoproteins. (**b**) Exogenous β-1,3-glucanases also contribute to improved wine quality and longevity by promoting the lysis of contaminant wild yeast, which are responsible for several off-flavors, and by hydrolyzing the extracellular β-glucan-rich slime produced by contaminant fungi and bacteria, which are the cause of the undesirable wine “ropiness”.

In the wine industry, commercial glucanases are especially used in the stabilization (clarification, filtration) and aging (maturation on lees) steps. During the winemaking process, the presence of lactic acid bacteria (mainly *Pediococcus* spp.) and the mold *Botrytis cinerea* can lead to a type of spoilage called ropiness. “Ropy” wines are a result of the production of exopolysaccharide slimes by these contaminating species, which are responsible for hampering clarification, causing filtration problems and leading to an unacceptable mouthfeel [260]. These slimes are mostly composed of high molecular weight β-1,3-glucan, which is why there are numerous commercial enzyme preparations containing β-glucanases, mostly from *Trichoderma* species, specifically produced to reduce the viscosity of musts and wine caused by these colloidal polysaccharides [261]. Other frequent wine contaminants are wild yeasts, which often lead to the development of off-flavors, decreasing wine quality. Since β-glucans are a major component of the yeast cell wall, β-1,3-glucanase supplementation is often used to control the development of wild species. A β-1,3-glucanase from *Delftia tsuruhatensis* has demonstrated the potential to both prevent slime production and undesirable yeast growth during vinification, improving the organoleptic properties of wine. This enzyme is particularly interesting because it is highly active under wine-relevant parameters, such as elevated ethanol, sulfite, and phenol concentrations, and at low pH values [262]. Certainly, one of the most interesting features of exogenous β-glucanase supplementation during vinification is its ability to improve the effects of the traditional “bâtonnage” technique, used during the “sur lie” (on lees) aging process. The “bâtonnage” consists in stirring the settled lees back into the wine, which are riddled with dead yeast. This allows the cellular constituents of starter yeasts, mainly mannoproteins, to be mixed with the wine. These mannoproteins, present in the cell wall of the yeasts, are associated with very positive quality and technological wine traits. Using β-glucanase supplementation, it is possible to exert a strong lysogenic action on the yeast, further promoting the release of mannoproteins into the wine (Figure 7). This post-fermentative treatment aims to improve the wine’s organoleptic properties by increasing its body, mouthfeel and creaminess, but it also reduces the risk of microbial spoilage, promotes protein and tartaric salt stabilization, reduces haziness, and improves the wine’s overall aging potential [261,263].

β-1,3-glucanases are also very useful tools in several biotechnological processes, such as the preparation of fungal cell protoplasts for fungal gene function studies [264], in the transformation and extraction of proteins in yeast protein expression systems [259], or even as part of biosensors for the detection of laminarin [265]. In summary, the potential applications of β-1,3-glucanases are many, which is a reflection of their multiple roles in biology, in addition to the importance of their natural substrates.

## 6. Final Remarks

β-glucans are a diverse class of complex polysaccharides synthesized by multiple organisms across many taxa. There are extensive structural differences between β-glucans, mostly as a result of their distinct sources, but also due to the extraction and purification methods used to obtain them. These differences have profound implications for the physicochemical and functional profiles of β-glucans. There is an abundance of literature attesting to the many applications of β-glucans, with particular emphasis on their therapeutic potential, which varies from antitumor and immunomodulatory effects, to improved glycemic control, cholesterol-lowering and probiotic properties. The multiple registered clinical trials using β-glucans for various pathologies are a clear testament to a longstanding interest in these compounds. Nonetheless, there are still significant barriers to their clinical use. The main issue is that the true extent of the relationship between molecular structure and functional profile remains to be clarified. Despite the many published papers on the biological activities of β-glucans, comparing results is often difficult, as the majority of authors used polysaccharides from different sources, with different sizes and, therefore, different physicochemical properties. It is then imperative to provide further characterization of β-glucans, and to optimize isolation and purification procedures, so that a proper structure–function relationship can be established. Only then will it be possible to identify the most promising compounds for clinical testing. β-glucanases, in addition to having enormous potential for application in many industrial and biotechnological processes, are an invaluable tool to study the structure of β-glucans. By having prior knowledge of a β-glucanase mechanism of action, it is possible to infer about a polysaccharide structure by analyzing the products of its hydrolysis. In turn, a proper understanding of a β-glucanase’s mechanism of action requires the use of a properly characterized substrate. The two are intrinsically connected, and their full biological and biotechnological potential can only be unlocked with systematic, methodical and comparable structure–function studies.

## Figures and Tables

**Table 1:** Distribution of characterized β-1,3-glucanases across glycoside hydrolase (GH) families and taxa, according to the CAZy.org database (as of February 2022).

β-1,3-glucanase Class	Domain	GH Families													
		3	5	8	9	16	17	51	55	64	81	128	152	157	158
EC 3.2.1.58	Bacteria		2						2						
	Fungi		15						13						
	Plants		2												
EC 3.2.1.39	Archaea					1									
	Bacteria		1			27	3		1	5	3	3		1	3
	Fungi					2	1		2		10	1	1		
	Plants						28				1				
	Animals					14									
EC 3.2.1.6	Bacteria	3		2	2	11		1							
	Fungi					6									
	Unclassified			3											

## Data Availability

Not applicable.
